# Complementary Effect of an Educational Website for Children and Adolescents with Primary Headaches in Tertiary Care: A Randomized Controlled Trial

**DOI:** 10.3390/children12060716

**Published:** 2025-05-30

**Authors:** Henrike Goldstein, Lisa-Marie Rau, Verena Bachhausen, Julia Wager

**Affiliations:** 1 German Paediatric Pain Centre, Children’s and Adolescents’ Hospital Datteln, 45711 Datteln, Germany; h.goldstein@deutsches-kinderschmerzzentrum.de (H.G.); l.rau@deutsches-kinderschmerzzentrum.de (L.-M.R.); v.bachhausen@deutsches-kinderschmerzzentrum.de (V.B.); 2 Department of Children’s Pain Therapy and Paediatric Palliative Care, Faculty of Health, School of Medicine, Witten/Herdecke University, 58455 Witten, Germany

**Keywords:** headache, pediatrics, outpatients, health communication, digital health, web browser, multi-level analysis

## Abstract

**Background/Objectives**: Tension-type headache and migraine are common among children and adolescents, often causing significant distress and persisting into adulthood. While outpatient pain therapy is essential, it is not always sufficient. To enhance initial therapy consultations, we evaluated a new educational website in a pediatric outpatient pain clinic. **Methods**: Ninety-three children with headache (*M*_age_ = 12.66, *SD*_age_ = 2.86) visiting a specialized tertiary care center were randomly assigned to either an intervention or control group. The intervention group received immediate access to the website, while the control group was given access after the final assessment. Three online follow-up assessments occurred at four-week intervals after baseline. Recruitment occurred between April 2021 and October 2022. **Results**: Headache-related disability, headache days, and days with headache medication use significantly decreased over time (main effect; disability: *β* = −0.23, 95%-CI = [−0.36; −0.09], *p* = 0.001; days: *β* = −0.18, 95%-CI = [−0.32; 0.03], *p* = 0.018, medication: *β *= −0.16, 95%-CI = [−0.31; −0.02], *p* = 0.026). No statistically significant changes were observed for average headache intensity, passive pain coping, positive self-instructions, seeking social support, pain self-efficacy, and headache-related knowledge. Groups did not differ in their improvement over time (interaction effect). Per-protocol analysis yielded a similar trend: headache-related disability improved significantly with no interaction effects. Despite the limited impact on headache management, children rated the website as relevant and easy to understand. **Conclusions**: While well-received, the website’s effectiveness may have been limited by participants’ prior knowledge, concurrent therapies, and low engagement. Future research should focus on better integrating the tool into treatment plans, optimizing usage, and tailoring content to varying knowledge levels. Nevertheless, it shows potential as a long-term self-management tool.

## 1. Introduction

Headaches in children and adolescents are widespread. According to two recent studies from Germany, about one in three children has headaches at least once a month [1] and one in five experiences weekly headaches for at least three months [2]. Migraine and tension-type headache are the most common headache types in children [3]. These two primary headaches differ regarding symptomatology [4] and treatment recommendations [5].

In addition to the high level of suffering, children and adolescents with severe primary headaches often participate little in everyday life and face increased risks of school absenteeism [6], frequent doctor visits [7,8], and non-indicated medication use, which can evolve into medication-induced headache [9]. Recurrent headaches often persist into adulthood, leading to increased costs for both society and the healthcare system [10,11]. Outpatient healthcare services play an essential role in the management of recurring pain, offering accessible and effective treatments that can significantly reduce symptom severity and disability in everyday life [12,13].

However, even specialized outpatient treatments are not fully effective for all patients. This is exemplified in a study by Hechler et al. (2011)—after 12 months, 11% of the participating children had transitioned to more intensive treatment, 12% remained in outpatient care, and about 30% reported continued difficulty attending school regularly [14]. This highlights the need for alternative care approaches or complementary elements to enhance the effectiveness of outpatient treatment.

Education on headaches can be a valuable tool to better understand their biopsychosocial origin, guide appropriate treatment strategies, and help prevent symptom worsening [15]. It also provides confidence in dealing with pain [16] and strengthens self-reliance [17]. This is essential to create the basis for good self-management. The internet is a crucial resource for children and adolescents seeking health information; however, young people often lack the knowledge and experience to search effectively or evaluate the reliability of sources [18]. We have thus created an accessible website to provide the most important information and guideline-compliant treatment advice on the two most common primary types of headache in children and adolescents: tension-type headache and migraine (www.meine-kopfsache.com; also available in English: https://www.headeggs.org) (accessed on 24 April 2025) [15].

The current study aims to investigate whether providing complementary access to the website improves the results of outpatient headache treatment. This research question is addressed in a randomized controlled trial (RCT) in which pediatric patients with primary headaches (migraine and/or tension-type headache) receive treatment as usual in an outpatient pain clinic and the intervention group (IG) additionally receives access to the website along with quizzes. It has been shown that pediatric pain therapy—especially when followed by long-term support—can improve pain-related disability, pain intensity, and pain self-efficacy [19]. Moreover, the website used in the current study has previously been shown to significantly increase headache-related knowledge and reduce passive pain coping strategies in a school sample [20]. We aimed to examine these effects in the present study as well. Thus, we hypothesized that the IG would show stronger improvements in headache-related disability (primary outcome), headache-related knowledge, pain self-efficacy, headache-related behavior, and headache characteristics (secondary outcomes).

As both the symptoms and the treatment recommendations for migraine and tension-type headaches differ greatly, we further wanted to investigate whether patients with and without a migraine diagnosis experience the website differently. Lastly, we compared the self-reported website visits of both groups to explore differences in visiting behavior and satisfaction.

## 2. Materials and Methods

### 2.1. Design

Data were collected from April 2021 to October 2022 in the pain outpatient department of the German Paediatric Pain Center, Datteln, Germany, as part of the CHAP project (“Chronic headache in adolescents: The patient perspective on health care utilization”, Reference Number: 01GY1615). The current RCT was preregistered in the German Clinical Trials Register (ID: DRKS00024370). It consisted of four measurement points, in which patients completed questionnaires at four-week intervals. The first assessment took place in the outpatient pain clinic on tablet computers, while the rest were completed via an emailed link to an online questionnaire.

### 2.2. Procedure

Eligibility criteria included (1) sufficient German language proficiency, (2) recommendation for outpatient pain therapy for primary headaches, (3) between 8 and 18 years old, and (4) informed assent/consent from both patients and parents.

The first questionnaire was included in a standard diagnostic questionnaire that all patients at the recruiting outpatient facility complete before their initial appointment. During the appointment, patients received a diagnosis along with recommendations as to their next steps, such as continuing outpatient or inpatient therapy, or taking other measures first, such as further diagnostics or outpatient psychotherapy. We selected outpatient pain therapy recommendation as an eligibility criterion based on the assumption that this group and their headaches would be most responsive to the intervention. After the appointment, patients who met the eligibility criteria were invited to participate in the study.

After giving consent/assent, participants were randomly assigned to either the IG or control group (CG) stratified by gender. The random allocation sequence was generated using R (R Foundation for Statistical Computing, Vienna, Austria). Group assignment was conducted by the research team using sealed envelopes. Blinding was not feasible. The distribution for the first five months was a 1:1 ratio. After six months, however, it became clear that the response rate in the IG was noticeably lower. As a result, the allocation ratio was adjusted to a 2 IG:1 CG ratio (see power calculation). Immediately after their appointment, patients in the IG received a flyer with the website address (www.meine-kopfsache.com, English website: https://www.headeggs.org) (accessed on 24 April 2025) and a QR-code leading to it, as well as printed quizzes on website content. With website access, children and their families could explore the content independently from home. The quiz questions guided users through the website step-by-step, encouraging a thorough exploration of its content. Patients in the CG received access to the website via email after completing all assessments. 

The primary goal of the website was to teach users how to distinguish between tension-type headache and migraine (the main topic of the landing page) and how best to manage each type of headache (the two subsequent pages). The pages concerning different headache types were divided into sections that explained headache characteristics, how they develop, and what can be done to treat them. Additional pages outlined strategies to reduce headache frequency, particularly topics such as exercise, stress management/relaxation, and sleep. Critical information was provided in both video and text form so users could choose their preferred adaption mode. Additional content was hidden in unfolding sections to avoid overwhelming users with text. To better assess individual situations, there were checklists at various points where users could mark pertinent statements, along with feedback on whether any action was needed—for example, regarding better sleep hygiene. This was intended to encourage a more active lifestyle. There were also various interactive elements, such as clickable buttons with responses, like a “slot machine” that generated movement ideas, or sliders that illustrated changes in migraine intensity with or without medication use.

Overall, the aim was to empower children to actively manage their headaches through increased knowledge and to strengthen their pain self-efficacy. This was purposed to have an impact on their headache-related disability and headache-related behavior and thus the headache characteristics. Beyond knowledge transfer, the website was also intended as a resource that remains available after the outpatient appointment, providing long-term support and information.

The interactive website was developed by headache specialists from the German Paediatric Pain Centre in collaboration with patients on behalf of the self-help group UVSD SchmerzLOS e.V., Neumünster, Germany, with financial support from the Techniker Krankenkasse health insurance fund, Hamburg, Germany. These organizations did not have any influence on the website’s content. The website was not visible to search engines before or during the study period. For more information on the website, see Goldstein et al. [20].

### 2.3. Sample

**Figure 1:**
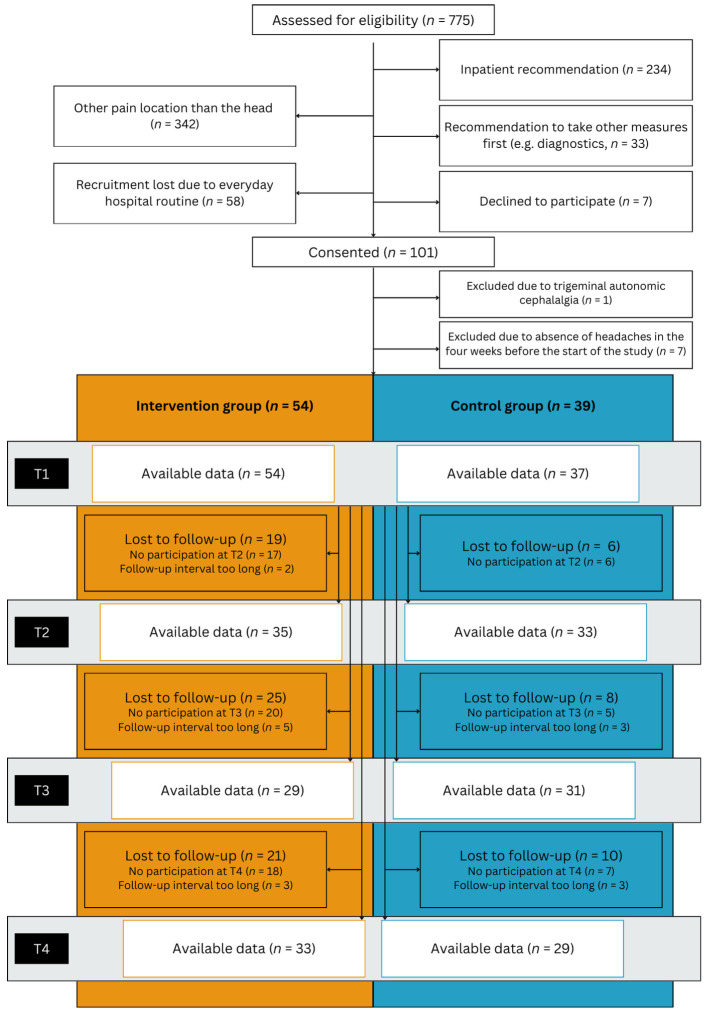
Flowchart. T1, T2, T3 and T4; First, second, third, and fourth assessments. Partly completed: IG: *n*_T1_ = 2, *n*_T2_ = 3, *n*_T4_ = 2. CG: *n*_T3_ = 2. T1 data of two children were removed from T1, as they answered the questionnaire after their appointment.

*N* = 775 children were of the required age and completed the standard diagnostic questionnaire (Figure 1). Of these, *n* = 108 fulfilled eligibility criteria 1 to 3. Of these families, *n* = 7 did not agree to participate.

In total, 101 families consented to participate in the study. Four families did not complete any further questionnaires after baseline, and one family discontinued after T2; however, the data they had provided up to that point were included in the analysis. Seven children reported in the initial questionnaire that they had not experienced any headaches in the past four weeks; these cases were excluded from the analysis, as no further improvement could be expected from the intervention. Additionally, one child was excluded because his type of headache, trigeminal autonomic cephalalgia, was not addressed on the website. Of the 93 included participants, 54 were assigned to the IG and 39 to the CG (66.67% girls; *M*_age_  = 12.66 years, *SD*_age_  = 2.86 years, *range*_age_ = 8–17 years). To assess the success of randomization, baseline differences between the IG and CG were evaluated using Welch’s *t*-test (continuous variables) and chi-squared tests (χ^2^, categorical variables; Table 1). There were no statistically significant differences between participants in the IG and the CG in terms of demographic, headache-related, or psychological characteristics (all *p*-values > 0.05). According to the diagnoses extracted from clinical documentation, 66 of the 93 children had a migraine diagnosis (70.97%). Follow-up assessment data were excluded from analysis if too much time had elapsed between their completion dates—specifically, more than 53 days between T1 and T2, 83 days between T1 and T3, or 113 days between T1 and T4. See Figure 1 for the participant flowchart and Table 1 for a detailed sample description at T1.

### 2.4. Measures

Patients self-reported their gender at baseline. We calculated the age at the initial visit based on the date of birth, which was available from clinical documentation. All patients indicated where they had experienced pain in the past four weeks.

#### 2.4.1. Headache-Related Disability

Our primary outcome was headache-related disability at the last assessment, T4. It was assessed using a modification of the Pediatric Migraine Disability Assessment (PedMIDAS). In its original version, this refers to disability in the past three months [21]. We used an adapted version that asks about disability in the past four weeks. This not only suits the 4-week assessment interval but has also shown improved recall accuracy compared to the 3-month version [22].

The PedMIDAS questionnaire consists of 6 questions on how many days in the specified period one was unable to carry out an activity or could only do so with limited ability. The numeric response was limited to 30 days (20 days for the questions relating to school days). The responses are summed to a total score with a possible scale range of 0–160. Disability is classified as little to none (scores between 0 and 10), mild (11–30), moderate (31–50), and severe (>50) [23]. The internal consistency in the current sample was acceptable to high (T1: α = 0.70; T2: α = 0.88; T3: α = 0.81; T4: α = 0.82). To assess the clinical relevance of disability, participants reported the number of days they experienced headaches over the past four weeks (maximum 30 days). We also asked about the average headache intensity during this time, rated on a Numeric Rating Scale (NRS) ranging from 0 = no pain to 10 = strongest pain. These factors do not contribute to the sum score.

#### 2.4.2. Headache-Related Knowledge

Headache knowledge was assessed using a 12-item questionnaire specifically developed for the website content. The questionnaire was validated in *N* = 793 students showing sound psychometric properties according to item response theory [20]. Knowledge scores may range from 0 to 12, with each correct answer contributing one point to the total score. Headache-specific knowledge was not part of T3 to minimize testing effects [24,25] and participant burden.

#### 2.4.3. Pain Self-Efficacy

All children rated their pain handling using the Scale for Pain Self-Efficacy (SPaSE) [26]. This instrument comprises 11 items (e.g., I’ve got my pain under control), with responses recorded on a five-point scale (0 = not true to 4 = true; higher scores reflect greater self-efficacy). The internal consistency of the scale was high to excellent in this sample (T1: α = 0.87; T2: α = 0.91; T3: α = 0.89; T4: α = 0.92).

#### 2.4.4. Headache-Related Behavior

The participants were asked about their pain coping strategies using the Pediatric Pain Coping Inventory—revised (PPCI-r [27]). It consists of three subscales: passive pain coping (10 items; internal consistency acceptable to high: T1: α = 0.75; T2: α = 0.77; T3: α = 0.77; T4: α = 0.82), positive self-instructions (7 items; internal consistency questionable to high: T1: α = 0.68; T2: α = 0.76; T3: α = 0.82; T4: α = 0.83), and seeking social support (8 items; internal consistency high: T1: α = 0.85; T2: α = 0.84; T3: α = 0.80; T4: α = 0.87)— with the response options 0 = almost never, 1 = sometimes, and 2 = often. A sum score was calculated for each subscale.

To assess medication intake, we asked for the number of days in the past four weeks the participants took medication for their headache (maximum 30 days).

#### 2.4.5. Feedback on the Website

In assessments T2–T4, children of the IG were asked whether they had visited the website in the past four weeks. If they reported having done so, they were asked for feedback on it. This included their overall satisfaction (“What did you think of the website?”; 1 = very good, 6 = insufficient), the relevance of the website content (0 = not important at all, 4 = very important), and the comprehensibility of the information provided (0 = not comprehensible at all to 4 = very comprehensible). The question on overall satisfaction was recoded for better comparability to the other two items (−1 = insufficient, 4 = very good). Higher numbers thus indicate greater satisfaction.

To detect treatment contamination, patients in the CG were asked at T4 if they had ever heard of and/or visited the website.

### 2.5. Power Calculation

The sample size was calculated for a repeated measures ANOVA with a within-between interaction. To detect a moderate effect (f = 0.5 [28]) with 90% power and an alpha error probability α < 0.05, *N* = 60 patients had to be included. Therefore, our aim was to have 30 complete data sets in each group at the primary endpoint T4. With an expected dropout rate of 30%, *N* = 78 patients had to be recruited. When it became evident that the participation rate at follow-ups was lower than anticipated, recruitment was extended to include 100 children. Thus, 29 (CG) and 31 (IG) complete data sets at T4 could be included in the analyses.

### 2.6. Data Analyses

The statistical significance threshold was set at *p* = 0.05. For group comparisons and post-hoc tests, *p*-values were adjusted using the Benjamini–Hochberg correction and presented alongside the unadjusted *p*-values [29]. Effect sizes were interpreted based on Cohen’s guidelines [28]. Data processing was conducted in SPSS, IBM Germany GmbH, Böblingen, Germany, while analyses were performed using R and RStudio (Posit PBC, Boston, MA, USA, see Supplementary Material S1 for the R packages used).

#### 2.6.1. Multiple Imputation of Missing Data

To address missing data (25.4%), we conducted all multilevel analyses using multiply imputed datasets with predictive mean matching (30 datasets, 20 iterations, predictors with τ > 0.1; R package mice). Consequently, all reported coefficients represent pooled estimates. This approach was chosen as it typically yields less biased results compared to listwise deletion [30].

#### 2.6.2. Intention-to-Treat Analysis

To assess the impact of the intervention (in addition to the regular outpatient treatment), we conducted multilevel models including all participating patients as an intention-to-treat analysis. They were performed for all variables measured on a continuous scale, accounting for the hierarchical structure of the data (with observations nested within patients). These models employed an autocorrelative covariance structure and utilized restricted maximum likelihood estimation and Kenward Roger approximation [31] (R package nlme).

Uncertainty intervals (equal-tailed) and *p*-values (two-tailed) were estimated using a Wald *t*-distribution approximation. The intraclass correlation coefficients ranged from 0.16 to 0.70, demonstrating sufficient between-person variance to warrant the use of multilevel modeling. In the multilevel models, time was treated as a continuous variable, while for post hoc analyses, it was treated as categorical. For categorical time, follow-up assessments (T2–T4) were each compared to the reference category T1. The CG was used as the reference category for the group variable. The time × group interaction assesses the intervention effect reflected in a divergent progression of the two groups over time. Each model included baseline values (T1) to obtain more precise estimates, assuming no systematic differences between groups due to successful randomization [32].

#### 2.6.3. Per-Protocol Analysis

A per-protocol analysis was conducted to analyze how the intervention worked when implemented as intended. The per-protocol sample consisted of patients who met the following criteria: (a) they completed T2, and (b) they were either in the IG and reported at T2 that they had visited the website or in the CG and reported at T4 that they had never visited the website. T2 was selected as the critical time point for IG participants to have accessed the website, as it is assumed that engagement with the website would influence all subsequent measurements.

#### 2.6.4. Efficacy of the Website Depending on Migraine Diagnosis

To examine whether intervention effectiveness differed for children with and without migraine, we added migraine diagnosis as a third factor in the multilevel models. We compared children who had received a migraine diagnosis at the initial appointment to those without, with the latter forming the reference category in a three-way interaction of time × group × migraine.

#### 2.6.5. Website Usage

We also investigated whether website use fluctuated over time and depended on the presence of a migraine diagnosis. As multilevel modeling was not feasible due to insufficient intra-person variance, we calculated a generalized linear model in which the binary, self-reported variable of whether the website had been visited in the past four weeks was linked to the presence of a migraine diagnosis (time × migraine, absence of migraine diagnosis set as a reference category). We also explored descriptively whether the self-reported visits to the individual pages of the website (landing page, Tension-Type Headache, Migraine, Sleep, Movement, Stress) differed depending on having a migraine diagnosis. For these analyses, non-imputed data were used.

### 2.7. Ethics

The study was approved by the Ethics Committee of Witten/Herdecke University (reference number 268/2020, 16 February 2021).

## 3. Results

### 3.1. Effectiveness of the Website-Based Education

#### 3.1.1. Results of the Intention-to-Treat Analysis

**Figure 2:**
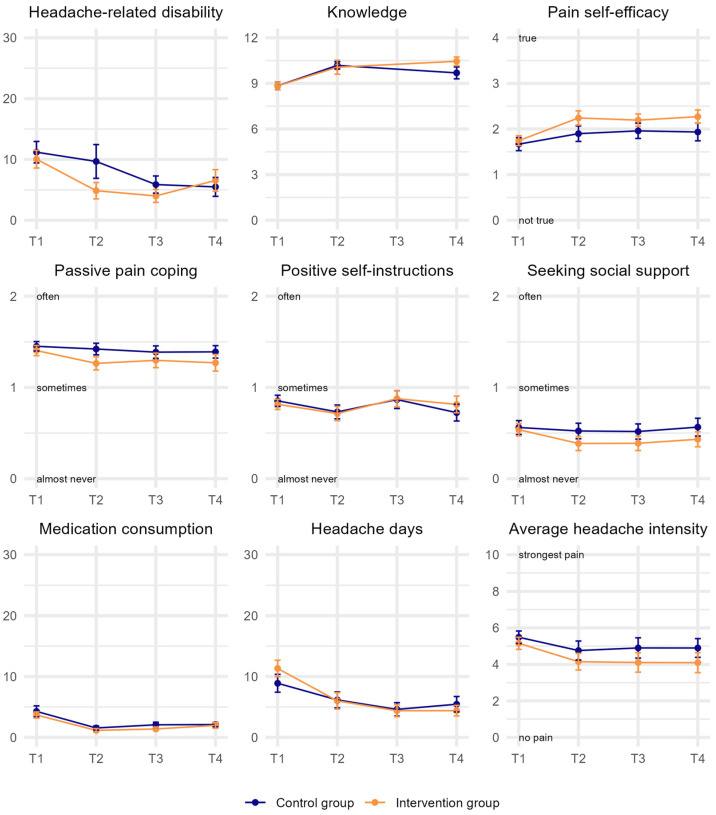
Mean trajectories of outcomes stratified by group (intervention vs. control group). Assessments occurred before the appointment (T1), and 4, 8, and 12 weeks after T1 (T2–T4). Error bars represent 95% confidence intervals. *N* = 93.

Headache-related disability decreased over time, resulting in a main effect of time (*β* = −0.23, 95%-CI = [−0.36; −0.09], *p* = 0.001, Table 2). Post hoc tests showed that, across groups, days with headache-related disability significantly decreased by 4.83 days from T1 to T3 (*d* = −0.38, Figure 2, Supplementary Material S2) and by 4.04 days from T1 to T4 (*d* = −0.37). A statistically significant interaction effect of time × group was not detected.

Headache days significantly decreased over time (*β* = −0.18, 95%-CI = [−0.32; 0.03], *p* = 0.018), with significant differences of 5.35 days between T1 and T3 and 5.40 days between T1 and T4. Mean headache intensity did not change significantly. Also, there were no significant interaction effects regarding either of these headache characteristics.

For the three subscales of the PPCI-r (passive pain coping, positive self-instructions, and seeking social support), pain self-efficacy, and headache-related knowledge, no statistically significant main effects or interaction effects were observed. The number of days with medication use for headaches decreased significantly by 2.21 days from T1 to T3 and by 1.87 days from T1 to T4 (*β *= −0.16, 95%-CI = [−0.31; −0.02], *p* = 0.026); however, no significant interaction effect was detected.

#### 3.1.2. Results of the Per-Protocol Analysis

The per-protocol analysis included 51 children who completed the T2 assessment in full: 23 from the IG who reported having visited the website at T2, and 28 from the control group who reported having never accessed it. Analyses revealed a significant main effect of time for days with headache-related disability (*β *= −0.17, 95%-CI = [−0.32; −0.02], *p* = 0.023, Supplementary Material S3), with a decrease of 3.22 days from T1 to T2, 4.83 days from T1 to T3 and 3.40 days from T1 to T4. The categorical post hoc tests did not reach statistical significance. No statistically significant effects were observed for the other examined outcomes.

### 3.2. Migraine as a Predictor

Including migraine as a third factor in the multilevel models did not reveal statistically significant interaction effects of time × group × migraine for the investigated outcomes (Supplementary Material S4). They occurred neither in the intention-to-treat nor in the per-protocol analysis. The additional provision of the website was therefore not differently effective regarding the considered outcomes for children with a migraine diagnosis compared to those without this diagnosis. A main effect of migraine was detected concerning passive pain coping strategies, with children with migraine diagnosis showing higher scores (*β* = 0.61, 95%-CI = [0.16; 1.06], *p* = 0.008). Across assessments and intervention groups, the mean for children with migraine diagnosis was 14.97 (*SD* = 3.39) and for those without this diagnosis, it was 10.49 (*SD* = 3.74).

### 3.3. Use and Feedback on the Website

At T2, the website was visited by 23 of the 32 responding children in the IG (71.88%). They rated the website with an average of 3.35 (*SD* = 0.65), which corresponds to a value between very good and good. At T3, 12 out of 29 (41.38%) participating patients in the IG visited the website. They gave an average score of 3.33 (*SD* = 0.78), and at T4, the 10 children who participated and had looked at the website in the past four weeks gave a score of 3.1 (*SD* = 1.20).

At T2, most children reported having looked at the landing page addressing distinguishing tension-type headache from migraine (19 of 23, 82.61%), followed by the pages on tension-type headache (78.26%) and migraine (47.83%). Fewer children reported having looked at the pages on stress (21.74%), exercise (30.43%), or sleep (26.09%). When asked how important and how comprehensible they considered the website to be, the children reported mean values of 3.17 (*SD* = 0.58) and 3.70 (*SD* = 0.56) at T2, which corresponds to a rating between rather important and very important or rather comprehensible and very comprehensible.

**Figure 3:**
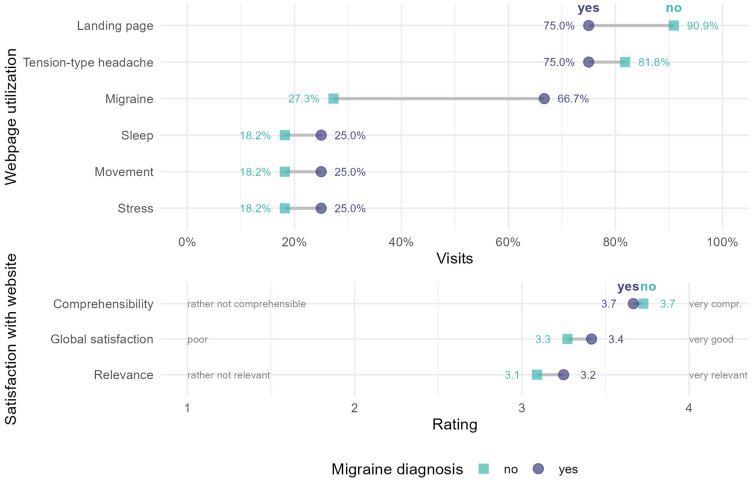
Utilization of and satisfaction with the website at the second assessment (T2) by group (presence vs. absence of a migraine diagnosis). Circle: migraine diagnosis (*n* = 12); Square: no migraine diagnosis (*n* = 11).

A comparison of patients at T2 with and without a migraine diagnosis regarding the utilization of the individual webpages and feedback on the website can be found in Figure 3. The pages on the differentiation of headache types and on tension-type headache were accessed most frequently and slightly more by patients without a migraine diagnosis than by those with a migraine diagnosis. The greatest deviation can be found in the number of visits to the page on migraine, which was used more often by children with a migraine diagnosis. The pages on health-promoting behavior (sleep, exercise, and stress) were used slightly more by children with migraine but less often overall than the other pages. Children with a migraine diagnosis rated the website in general and the relevance of its content slightly more positively than children without this diagnosis.

The generalized linear model predicting website visits based on time and migraine diagnosis detected a significant main effect of time (OR = 0.28, 95%-CI = [0.09; 0.71], *p* = 0.013). Thus, from assessment to assessment, the likelihood that the website would be accessed decreased by 0.28. However, neither a statistically significant main effect for migraine nor an interaction effect was observed (migraine: OR = 0.08, 95%-CI = [0.00; 2.75], *p* = 0.179; interaction effect migraine × time: OR = 1.92, 95%-CI = [0.60; 6.88], *p* = 0.286). This means that the change in website usage did not differ significantly between patients with or without a migraine diagnosis.

## 4. Discussion

In this RCT, we investigated the effects of an educational headache website on headache-related disability, knowledge about headaches, pain self-efficacy, and headache-related behaviors. This intervention was provided in addition to an initial consultation at the outpatient pain department of a pediatric clinic. We also explored how children and adolescents perceive the website.

Overall, our primary outcome, headache-related disability, significantly decreased over time across the sample. Reductions were also observed in the number of headache days and the use of headache-related medications. However, the trajectories between the IG and CG did not differ significantly, and thus no intervention effect was detected. A per-protocol analysis, which included only children who completed the follow-up questionnaire and either actively engaged with the website (IG) or did not (CG), also revealed a significant decrease in headache-related disability but no significant interaction effects. The website’s effectiveness furthermore did not differ between patients with and without migraine diagnosis. Nevertheless, patients rated the website as good, relevant, and highly comprehensible. They reported having visited the sections on distinguishing between different types of headaches and on specific headache types (tension-type headache and migraine) more frequently than the pages focused on healthy lifestyle topics such as exercise, stress management/relaxation, and sleep.

A potential explanation for the lack of significant intervention effects is the high level of knowledge about headache. On the one hand, this concerns prior knowledge, indicated by a mean headache-related knowledge score of 8.84 out of 12 (*SD* = 1.70) at baseline, considerably higher than the 6.3 (*SD* = 1.9) reported in a school-based study using the same website, where a significant increase in knowledge was observed [20]. This difference in prior knowledge likely exists because children who seek an appointment at the pain clinic have been dealing with the issue for a longer time and have already acquired more knowledge compared to those in a general population sample. Furthermore, the recruitment took place after a comprehensive initial consultation at the outpatient pain clinic, where both IG and CG received an in-depth educational session as part of the headache therapy, potentially covering some of the website’s content. It should also be considered whether the questionnaire was too simple for this sample. A ceiling effect is typically present when around 15% of respondents achieve the maximum score [33]. In this case, the percentages were 3.3% at T1, 29.1% at T2, and 22.7% at T4, indicating a possible ceiling effect at later time points. Due to this ceiling effect, values above the threshold cannot be observed, resulting in one-sided data censoring. This can reduce variance estimates and cause the recorded mean to be lower than the true mean [34]. Future research should consider tailoring interventions based on baseline knowledge levels to offer participants information that is new and relevant to them. It might also be worthwhile to include more challenging questions in the questionnaire to better map higher levels of knowledge accurately.

Another factor that may explain these findings is the frequency and depth of engagement with the website. Previous studies on computer-based interventions for children and adolescents with recurrent headaches were able to positively influence headache-related outcomes, such as headache severity or headache frequency [35]. However, these interventions went beyond simply providing a resource—they required active participation in a structured, intensive program. For example, Rapoff et al. provided a CD-ROM program as a supplement to the treating neurologist’s recommendations and prescriptions [36]. Children were to complete the program within four weeks, working through approximately one lesson per day and taking simple quizzes to assess their understanding of the material. The focus shifted weekly, covering topics like cognitive-behavioral treatments, relaxation techniques, problem-solving, stress management, pain behavior, and parental responses to pain. They observed significant reductions in headache-related disability, as measured by the PedMIDAS, at a 3-month follow-up, as well as a post-intervention reduction in headache severity among children with migraine. It appears that their analyses included only participants who were motivated to complete the full therapy program. Our program was significantly less intensive, engagement was monitored less closely, and the criteria for inclusion in our per-protocol analysis were more lenient. Trautmann et al. achieved significant reductions in headache frequency and duration through an internet-based self-help training program for children and adolescents with recurrent headaches [37]. Their program spanned six weeks and included internet-delivered education, homework, and email contact with the therapist, as well as booster sessions at four and eight weeks after the end of the program. Contrasting to these programs, the web-based intervention used in the current study relied solely on voluntary engagement.

When evaluated in a school-based sample that included children with and without headaches, the website was explored collectively in the classroom and accompanied by a workbook to encourage intensive engagement with the material. A significant increase in knowledge and a reduction in passive coping strategies in favor of the IG were observed [20]. In the present study, however, it is unclear whether the children made use of the provided trivia questions at all. Previous research on e-learning environments has shown that higher student engagement leads to better academic performance [38], suggesting that the level of engagement with the website in this study could be a critical factor. Future studies on comparable websites should therefore contain clear tasks to engage with the website and thus maximize the interventional effect.

Another aspect that distinguishes the website evaluated in this study from the interventions of the other discussed studies is the absence of interactive feedback and dialogue features, such as the therapist email contact used in Trautmann et al. [37]. Features like these may help reinforce material through ongoing discussions, clarify ambiguities, and provide more personalized support. In contrast, the current study’s website includes knowledge checks with corrective feedback and self-assessments on physical activity, stress, and sleep habits to encourage reflection and develop better self-awareness. However, the platform does not offer content tailored to the individual, and recommendations remain general. Integrating features such as chatbots or direct contact with outpatient pain clinic therapists could enable more intensive and personalized care.

Parents were actively involved in the computer-based studies on children with recurrent headaches referenced earlier [36,37]. In the study by Rapoff et al., parents received information about the intervention, their supportive role (e.g., helping with homework and a headache diary), and an overview of the lessons. Similarly, Trautmann et al. had parents complete questionnaires on their child’s symptoms. Although parents in the present study attended the initial outpatient pain clinic appointment, those in the IG were neither specifically informed about the website’s parent-focused section nor explicitly encouraged to engage with it. The website might have been more effective if it had placed greater emphasis on parental involvement, which has long been recognized as an important factor in managing and influencing the course of pediatric pain [39].

We were furthermore unable to confirm our hypothesis that the website’s effectiveness differs between patients with and without a migraine diagnosis. Thus, the same limiting factors mentioned earlier appear to affect both groups equally. While the core content and key takeaways may vary between the two diagnosis groups, no differences in overall effectiveness were observed. The detected main effect of migraine, where children with a migraine diagnosis reported higher passive pain coping, may be explained by acute migraine attacks, which make physical activity challenging, and by treatment guidelines that recommend rest following migraine medication [40]. Additionally, the groups differed in their use of the different webpages. Children without a migraine diagnosis more frequently accessed pages related to tension-type headache, while those with a migraine diagnosis visited the corresponding migraine-related content. Despite this difference in content consumption, no significant differences were found regarding the effectiveness of the website.

Patients also reported visiting the educational pages on different types of headaches more frequently than those focused on maintaining a healthy lifestyle to reduce headache frequency. This could suggest a reluctance to change behavior. Alternatively, the children might have felt that these general health aspects were not relevant to them or that their headaches were so frequent that factors like sleep were not perceived as the primary issues requiring attention. More individualized support, such as during therapy sessions, may be needed to help children adopt healthier lifestyles that support their recovery.

### 4.1. Strengths and Limitations

This study is among the first to evaluate an educational website on tension-type headache and migraine as a supplement to multidisciplinary outpatient therapy for children and adolescents. However, it is important to consider why the response rate in the IG was lower than in the CG. One possibility is that patients felt overwhelmed with information after their initial consultation at the pain clinic or that they needed time to implement what they had already learned before engaging with additional resources. As a result, they may not have been as receptive to another educational tool, which led to fewer completed questionnaires. Future research should explore how to better integrate the website into treatment and improve user engagement. Another explanation could be that the CG saw their access to the website as a reward for continuous participation, while the IG received their “reward” from the beginning. Implementing incentives for participation could thus improve retention rates in both groups.

Another limitation of our study is that it was conducted at a single site, where the socioeconomic status of the families receiving treatment is relatively homogeneous [41]. As a result, the generalizability to the broader population of affected children and adolescents is limited. However, the distribution between the intervention and control groups should be balanced due to the study’s randomized controlled design.

A limitation to our operationalization of website usage is that we relied on patients’ self-reported website engagement in the past four weeks. Collecting and analyzing objective user data from the website would have enabled analyses of the intensity and frequency of engagement needed to achieve noticeable effects. Tracking website visits, however, would have required personalized logins, which could have posed a barrier to website use. As it stands, the website remains easily accessible and seems to meet the needs of young patients with headaches, as reflected in their positive feedback on its comprehensibility, relevance, and overall satisfaction.

### 4.2. Practical Implications

Our results suggest that simply providing access to the website is not sufficient—it should be integrated into existing care structures and its use actively guided to maximize its effectiveness. While patients already find the website helpful, its impact could be enhanced through clear instructions from therapists on its use and benefits. One practical approach would be to grant patients and their families access to the website before the initial consultation. This would allow them to familiarize themselves with basic information in advance, freeing up more time during the consultation for individualized questions and developing tailored strategies. Specific website content could then be recommended based on the patient’s individual needs, with reminders or structured tasks to encourage engagement and facilitate the practical application of what has been learned. By embedding the website into a broader multimodal pain therapy approach, potentially incorporating greater parental involvement and targeted reflection exercises, it could serve as an integral treatment tool to deepen understanding and support sustained behavior change.

## 5. Conclusions

This study explored the potential of an educational website as an adjunct to outpatient headache therapy for children and adolescents. While the website was well-received in terms of clarity, satisfaction, and relevance, its overall impact on headache management was limited. The initial high level of knowledge among participants, the intensity of treatment they were already receiving, and the need for more frequent or intensive engagement with the website may have played a role in the lack of stronger effects.

Future research should explore ways to better integrate the website into existing treatment plans, determine optimal usage frequency, and account for the varying levels of prior knowledge among participants to maximize its potential benefits. Despite its limitations, the website shows promise as a supplementary educational tool, and its role in the long-term self-management of headaches warrants further exploration.

## Figures and Tables

**Table 1:** Baseline characteristics of the Intervention Group (IG) and Control Group (CG).

	Total	CG	IG	*p*
	*N *= 93	*n *= 39	*n *= 54	
Age (years)	12.66 (2.86)	12.38 (2.87)	12.85 (2.86)	0.440
Gender (girl) *	62 (66.67%)	23 (58.97%)	39 (72.22%)	0.265
Headache diagnosis *^a^				
Migraine	66 (70.97%)	31 (79.49%)	35 (64.81%)	0.191
Tension-type headache	80 (86.02%)	33 (84.62%)	47 (87.04%)	0.977
Headache-related disability ^b^	10.52 (10.46)	11.19 (10.59)	10.04 (10.44)	0.613
Headache-related disability: grades *^b^				0.823
Little to no disability	23 (25.84%)	8 (21.62%)	15 (28.85%)	
Mild disability	34 (38.20%)	16 (43.24%)	18 (34.62%)	
Moderate disability	13 (14.61%)	5 (13.51%)	8 (15.38%)	
Severe disability	19 (21.35%)	8 (21.62%)	11 (21.15%)	
Headache days in the past four weeks ^b^	10.31 (9.38)	8.89 (8.80)	11.33 (9.73)	0.222
Average headache intensity (0–10) ^b^	5.29 (2.25)	5.49 (2.05)	5.15 (2.40)	0.484
Headache-related knowledge (0–12) ^c^	8.84 (1.70)	8.84 (1.52)	8.83 (1.83)	0.990
Pain self-efficacy (0–44) ^b^	18.84 (8.93)	18.35 (9.43)	19.19 (8.63)	0.669
Passive pain coping (0–20) ^c^	14.23 (3.69)	14.51 (3.10)	14.04 (4.06)	0.528
Positive self-instructions (0–14) ^c^	5.81 (2.75)	5.97 (2.58)	5.70 (2.88)	0.642
Seeking social support (0–16) ^c^	4.37 (3.81)	4.49 (3.63)	4.30 (3.97)	0.814
Days with headache medication consumption in the past four weeks ^c^	3.92 (4.50)	4.27 (5.46)	3.68 (3.75)	0.567

Notes. Cells contain means (standard deviations) for numeric, absolute (relative) frequencies for categorical variables (indicated with an *). χ^2^- and *t*-tests were applied as appropriate (R package compareGroups). For children without headaches, logical imputation was performed by setting scores to 0 for the following outcomes: PedMidas items, headache days, average headache intensity, and days with headache-related medication consumption. Possible scale ranges are provided in brackets for each variable. ^a^ A child may have both diagnoses. ^b^ Data available for *n* = 89 children, as 2 participants from the CG did not complete T1 and 2 from the IG abandoned the questionnaire during completion. ^c^ Data available for *n* = 91 children, as 2 from the CG did not complete T1.

**Table 2:** Results of multilevel models (intention-to-treat analysis).

Model	Standardized Coefficient (SE)		95% CI	*t*	df	*p*
Headache-related disability						
Time ME	−0.23	(0.07)	[−0.36; −0.09]	−3.35	200.59	**0.001**
Group ME	0.00	(0.13)	[−0.27; 0.27]	−0.01	57.88	0.994
Time × Group	0.12	(0.10)	[−0.06; 0.31]	1.31	145.75	0.192
Headache days in the past four weeks						
Time ME	−0.18	(0.07)	[−0.32; −0.03]	−2.40	167.61	**0.018**
Group ME	−0.10	(0.13)	[−0.37; 0.16]	−0.78	59.07	0.438
Time × Group	−0.13	(0.11)	[−0.34; 0.08]	−1.21	100.26	0.230
Average headache intensity						
Time ME	−0.09	(0.08)	[−0.35; 0.07]	−1.09	137.03	0.277
Group ME	−0.13	(0.14)	[−0.41; 0.15]	−0.94	62.67	0.351
Time × Group	−0.09	(0.11)	[−0.31; 0.13]	−0.82	120.00	0.412
Headache-related knowledge						
Time ME	0.10	(0.10)	[−0.09; 0.30]	1.05	73.32	0.297
Group ME	0.07	(0.13)	[−0.20; 0.33]	0.51	51.18	0.614
Time × Group	0.13	(0.12)	[−0.11; 0.38]	1.08	89.12	0.285
Pain self-efficacy						
Time ME	0.05	(0.06)	[−0.07; 0.18]	0.82	122.14	0.413
Group ME	0.16	(0.13)	[−0.09; 0.41]	1.25	61.49	0.215
Time × Group	0.04	(0.08)	[−0.12; 0.21]	0.51	120.04	0.614
Passive pain coping						
Time ME	−0.04	(0.07)	[0.17; 0.09]	−0.56	150.40	0.577
Group ME	−0.12	(0.13)	[−0.39; 0.14]	−0.93	58.59	0.357
Time × Group	−0.05	(0.09)	[−0.24; 0.13]	−0.59	108.22	0.559
Positive self-instructions						
Time ME	−0.03	(0.07)	[−0.18; 0.12]	−0.41	82.51	0.684
Group ME	0.15	(0.14)	[−0.13; 0.42]	1.05	49.52	0.300
Time × Group	0.08	(0.10)	[−0.12; 0.28]	0.78	69.13	0.435
Seeking social support						
Time ME	0.01	(0.05)	[−0.10; 0.12]	0.16	129.29	0.872
Group ME	−0.12	(0.11)	[−0.34; 0.10]	−1.07	61.44	0.288
Time × Group	−0.04	(0.07)	[−0.18; 0.10]	−0.55	123.47	0.581
Days with headache medication consumption						
Time ME	−0.16	(0.07)	[−0.31; −0.02]	−2.25	161.61	**0.026**
Group ME	0.02	(0.11)	[−0.20; 0.23]	0.14	64.29	0.890
Time × Group	0.04	(0.10)	[−0.15; 0.23]	0.44	151.35	0.662

Notes. Observations are nested within patients (*N* = 93). Assessments took place before the intervention (T1) and subsequently at 4-week intervals (T2–T4). Treatment groups were the intervention group (IG) and the control group (CG). Reference category was CG for treatment; analysis was conducted using multiply imputed datasets. *p* < 0.05 are set in bold. SE = standard error; CI = confidence interval; df = degrees of freedom; ME = main effect.

## Data Availability

Data and syntaxes used for this study are available upon request from the corresponding author (J.W.).

## Supplementary Materials

The following supporting information can be downloaded at: https://www.mdpi.com/article/10.3390/children12060716/s1, Supplementary Material S1. Used R Packages; Supplementary Material S2. Post-hoc tests of multilevel models; Supplementary Material S3. Results of multilevel models (per-protocol analysis); Supplementary Material S4. Results of multilevel model time × group × migraine (intention-to-treat analysis).

## Abbreviations

The following abbreviations are used in this manuscript:

RCT	Randomized-controlled trial
IG	Intervention group
CG	Control group
CHAP	Chronic headache in adolescents: The patient perspective on health care utilization
PedMIDAS	Pediatric Migraine Disability Assessment
NRS	Numeric Rating Scale
SPaSE	Scale for Pain Self-Efficacy
PPCI-r	Pediatric Pain Coping Inventory–revised
