# Supplementary material for: Complementary Effect of an Educational Website for Children and Adolescents with Primary Headaches in Tertiary Care: A Randomized Controlled Trial
Source: Children (Basel). 2025 May 30;12(6):716. doi: 10.3390/children12060716 (PMC12190991; doi:10.3390/children12060716)
Supplement: Supplementary file 1 [file children-12-00716-s001.zip › S4 Results of Multi Level Model Time x Group x Migraine 20250424.pdf]

children

MDPI

Table S4. Results of the multilevel model time × group × migraine (intention-to-treat analysis).

Model	Standardized Coefficient (SE)		95% CI	*t*	df	*p*
Headache-related disability						
Time ME	-0.15	(0.14)	[-0.42; 0.13]	-1.04	268.94	0.297
Group ME	0.26	(0.25)	[-0.24; 0.75]	1.02	71.97	0.310
Migraine ME	0.30	(0.22)	[-0.14; 0.75]	1.36	82.19	0.178
Time × Group	-0.02	(0.18)	[-0.37; 0.34]	-0.08	197.45	0.934
Time × Migraine	-0.10	(0.16)	[-0.42; 0.21]	-0.64	254.61	0.520
Group × Migraine	-0.33	(0.29)	[-0.92; 0.26]	-1.12	70.71	0.266
Time × Group × Migraine	0.19	(0.21)	[-0.22; 0.60]	0.92	201.24	0.356
Headache days						
Time ME	-0.34	(0.15)	[-0.64; -0.04]	-2.22	190.57	**0.028**
Group ME	-0.16	(0.24)	[-0.65; 0.32]	-0.67	70.62	0.504
Migraine ME	-0.01	(0.22)	[-0.46; 0.43]	-0.06	79.58	0.951
Time × Group	-0.35	(0.20)	[-0.74; 0.04]	-1.76	129.60	0.080
Time × Migraine	0.21	(0.18)	[-0.14; 0.55]	1.17	170.95	0.242
Group × Migraine	0.09	(0.28)	[-0.47; 0.65]	0.32	72.08	0.747
Time × Group × Migraine	0.38	(0.22)	[-0.05; 0.82]	1.74	156.49	0.084
Average headache intensity						
Time ME	-0.14	(0.16)	[-0.46; 0.17]	-0.89	242.58	0.374
Group ME	-0.05	(0.27)	[-0.59; 0.48]	-0.19	72.54	0.847
Migraine ME	0.06	(0.25)	[-0.44; 0.55]	0.23	78.77	0.820
Time × Group	-0.04	(0.20)	[-0.43; 0.36]	-0.18	194.58	0.858
Time × Migraine	0.07	(0.19)	[-0.30; 0.44]	0.36	190.18	0.719
Group × Migraine	-0.11	(0.32)	[-0.74; 0.52]	-0.35	70.67	0.726
Time × Group × Migraine	-0.07	(0.23)	[-0.53; 0.39]	-0.29	179.72	0.771
Headache-related knowledge						
Time ME	0.04	(0.18)	[-0.32; 0.40]	0.22	154.77	0.825
Group ME	0.17	(0.23)	[-0.29; 0.62]	0.73	74.32	0.469
Migraine ME	0.27	(0.21)	[-0.16; 0.69]	1.25	76.44	0.215
Time × Group	0.18	(0.22)	[-0.26; 0.62]	0.81	138.87	0.422
Time × Migraine	0.08	(0.21)	[-0.33; 0.49]	0.39	143.56	0.696
Group × Migraine	-0.10	(0.26)	[-0.62; 0.43]	-0.36	75.79	0.719
Time × Group × Migraine	-0.05	(0.26)	[-0.57; 0.46]	-0.21	131.08	0.837
Pain self-efficacy						
Time ME	0.17	(0.12)	[-0.08; 0.41]	1.34	208.41	0.181
Group ME	-0.19	(0.22)	[-0.64; 0.26]	-0.85	76.14	0.397
Migraine ME	-0.62	(0.21)	[-1.05; -0.19	-2.88	75.94	**0.005**
Time × Group	-0.10	(0.16)	[-0.41; 0.22]	-0.60	144.20	0.546
Time × Migraine	-0.14	(0.14)	[-0.43; 0.14]	-1.00	177.89	0.317
Group × Migraine	0.41	(0.27)	[-0.12; 0.94]	1.53	70.43	0.129
Time × Group × Migraine	0.18	(0.19)	[-0.20; 0.56]	0.94	121.64	0.348
Passive pain coping						
Time ME	-0.06	(0.14)	[-0.34; 0.21]	-0.45	186.50	0.656
Group ME	0.05	(0.25)	[-0.44; 0.54]	0.20	58.90	0.843
Migraine ME	0.61	(0.22)	[0.16; 1.06]	2.71	76.19	**0.008**
Time × Group	-0.04	(0.19)	[-0.41; 0.33]	-0.22	104.49	0.823
Time × Migraine	0.03	(0.16)	[-0.28; 0.34]	0.20	195.83	0.841
Group × Migraine	-0.15	(0.29)	[-0.74; 0.43]	-0.53	53.33	0.601
Time × Group × Migraine	-0.01	(0.22)	[-0.45; 0.43]	-0.06	96.35	0.954
Positive self-instructions						
Time ME	0.04	(0.13)	[-0.22; 0.30]	0.29	221.77	0.771
Group ME	0.26	(0.25)	[-0.25; 0.77]	1.03	65.39	0.308
Migraine ME	0.07	(0.23)	[-0.38; 0.52]	0.30	78.37	0.769
Time × Group	0.06	(0.19)	[-0.30; 0.43]	0.35	98.60	0.728
Time × Migraine	-0.09	(0.15)	[-0.39; 0.22]	-0.57	189.26	0.573
Group × Migraine	-0.16	(0.29)	[-0.75; 0.42]	-0.56	66.66	0.576
Time × Group × Migraine	0.00	(0.20)	[-0.40; 0.40]	0.02	132.46	0.985
Seeking social support						
Time ME	0.03	(0.10)	[-0.17; 0.24]	0.34	265.32	0.738
Group ME	-0.18	(0.20)	[-0.57; 0.22]	-0.89	79.34	0.378

Migraine ME	0.17	(0.19)	[-0.21; 0.54]	0.89	80.15	0.378
Time × Group	-0.13	(0.14)	[-0.40; 0.15]	-0.91	150.06	0.363
Time × Migraine	-0.03	(0.12)	[-0.27; 0.21]	-0.27	225.64	0.788
Group × Migraine	0.13	(0.23)	[-0.34; 0.59]	0.54	76.31	0.587
Time × Group × Migraine	0.13	(0.17)	[-0.21; 0.46]	0.75	120.68	0.454
Days with headache medication consumption						
Time ME	-0.20	(0.15)	[-0.49; 0.09]	-1.35	253.61	0.179
Group ME	0.25	(0.20)	[-0.15; 0.65]	1.25	77.37	0.214
Migraine ME	0.32	(0.19)	[-0.06; 0.69]	1.69	79.28	0.095
Time × Group	-0.03	(0.18)	[-0.39; 0.33]	-0.15	205.70	0.885
Time × Migraine	0.04	(0.17)	[-0.29; 0.37]	0.25	232.91	0.806
Group × Migraine	-0.30	(0.25)	[-0.79; 0.20]	-1.20	66.45	0.234
Time × Group × Migraine	0.12	(0.22)	[-0.31; 0.54]	0.54	187.87	0.591

Notes. Observations are nested within patients (N = 93). Assessments were conducted before the intervention (T1) and then at 4-week intervals (T2 – T4). The treatment groups consisted of the intervention group (IG) and the control group (CG). Children without a migraine diagnosis were compared to those with the diagnosis. The reference categories were the CG for treatment and no migraine diagnosis for the migraine comparison; T1 was compared to the reference categories T4 (overall treatment effect) and T2 (intervention effect). $p < .05$ are indicated in bold. SE = standard error; CI = confidence interval; df = degrees of freedom; ME = main effect.
