# Supplementary material for: Complementary Effect of an Educational Website for Children and Adolescents with Primary Headaches in Tertiary Care: A Randomized Controlled Trial
Source: Children (Basel). 2025 May 30;12(6):716. doi: 10.3390/children12060716 (PMC12190991; doi:10.3390/children12060716)
Supplement: Supplementary file 1 [file children-12-00716-s001.zip › S3 Multi level model PP_20250424.pdf]

Table S3. Results of multilevel models (per-protocol analysis).

Model	Standardized Coefficient (SE)		95% CI	*t*	df	*p*
Headache-related disability						
Time ME	-0.17	(0.07)	[-0.32; -0.02]	-2.30	123.63	0.023
Group ME	-0.05	(0.15)	[-0.34; 0.25]	-0.31	41.47	0.761
Time × Group	0.10	(0.11)	[-0.11; 0.32]	0.94	126.09	0.349
Headache days						
Time ME	-0.14	(0.08)	[-0.30; 0.02]	-1.78	108.19	0.078
Group ME	-0.19	(0.15)	[-0.50; 0.12]	-1.21	40.56	0.233
Time × Group	-0.17	(0.12)	[-0.40; 0.06]	-1.50	113.65	0.137
Average headache intensity						
Time ME	-0.11	(0.08)	[-0.27; 0.06]	-1.29	105.37	0.201
Group ME	0.07	(0.16)	[-0.25; 0.39]	0.44	38.33	0.662
Time × Group	0.01	(0.12)	[-0.23; 0.25]	0.05	107.25	0.961
Headache-related knowledge						
Time ME	0.10	(0.11)	[-0.11; 0.32]	0.96	62.76	0.339
Group ME	0.21	(0.16)	[-0.11; 0.53]	1.33	39.69	0.192
Time × Group	0.19	(0.15)	[-0.12; 0.50]	1.23	69.44	0.224
Pain self-efficacy						
Time ME	0.08	(0.06)	[-0.05; 0.20]	1.24	92.59	0.218
Group ME	0.19	(0.15)	[-0.11; 0.50]	1.29	37.47	0.206
Time × Group	0.05	(0.09)	[-0.14; 0.23]	0.53	85.27	0.600
Passive pain coping						
Time ME	-0.06	(0.06)	[-0.18; 0.07]	-0.85	106.08	0.394
Group ME	-0.24	(0.14)	[-0.53; 0.05]	-1.65	39.24	0.106
Time × Group	-0.04	(0.10)	[-0.25; 0.16]	-0.43	84.14	0.665
Positive self-instructions						
Time ME	0.05	(0.07)	[-0.09; 0.19]	0.67	78.87	0.508
Group ME	0.08	(0.16)	[-025; 0.41]	0.50	38.85	0.621
Time × Group	-0.01	(0.10)	[-0.21; 0.19]	-0.10	86.56	0.918
Seeking social support						
Time ME	-0.01	(0.05)	[-0.11; 0.09]	-0.19	115.08	0.847
Group ME	-0.20	(0.12)	[-0.44; 0.04]	-1.65	42.60	0.105
Time × Group	-0.10	(0.07)	[-0.24; 0.05]	-1.28	115.31	0.204
Days with headache medication consumption						
Time ME	-0.13	(0.09)	[-0.30; 0.04]	-1.53	109.23	0.129
Group ME	0.03	(0.13)	[-0.24; 0.29]	0.20	42.24	0.842
Time × Group	0.07	(0.12)	[-0.18; 0.31]	0.55	120.14	0.584

Notes. Observations are nested within patients. Patients were included in this analysis if they had completed T2 and reported at T2 that they had visited the website within the past four weeks (intervention group (IG), n = 23) or if they reported at T4 that they had never visited the website (control group (CG), n = 28). Assessments took place before the intervention (T1) and subsequently at 4-week intervals (T2 – T4). Reference category was CG for treatment; analysis was conducted using multiply imputed datasets. $p < .05$ are set in bold. SE = standard error; CI = confidence interval; df = degrees of freedom; ME = main effect.
