# Supplementary material for: Complementary Effect of an Educational Website for Children and Adolescents with Primary Headaches in Tertiary Care: A Randomized Controlled Trial
Source: Children (Basel). 2025 May 30;12(6):716. doi: 10.3390/children12060716 (PMC12190991; doi:10.3390/children12060716)
Supplement: Supplementary file 1 [file children-12-00716-s001.zip › S2 Post-hoc-tests of multilevel models-MICE ITT-20250424.pdf]

Table S2. Post-hoc tests of multilevel models

**Model**	***t***	***df***	***p***	***p adj***	***d***
Headache-related disability					
Time ME	-3.35	200.59	**0.001**		
Group ME	-0.01	57.88	0.994		
Time × Group	1.31	145.75	0.192		
Post-Hoc-Tests					
T1-T2	-1.03	219.71	0.306	0.306	-0.15
T1-T3	-2.35	250.37	**0.020**	**0.030**	-0.38
T1-T4	-2.62	208.93	**0.009**	**0.028**	-0.37
Headache days					
Time ME	-2.40	167.61	**0.018**		
Group ME	-0.78	59.07	0.438		
Time × Group	-1.21	100.26	0.230		
Post-Hoc-Tests					
T1-T2	-1.23	179.26	0.219	0.219	-0.26
T1-T3	-2.07	196.76	**0.039**	0.118	-0.42
T1-T4	-2.00	189.54	**0.047**	0.071	-0.30
Days with headache medication consumption					
Time ME	-2.25	161.61	**0.026**		
Group ME	0.14	64.29	0.890		
Time × Group	0.44	151.35	0.662		
Post-Hoc-Tests					
T1-T2	-4.54	210.35	**< 0.001**	**< 0.001**	-0.76
T1-T3	-3.26	242.82	**0.001**	**0.001**	-0.57
T1-T4	-3.49	149.86	**0.001**	**0.001**	-0.53

Notes. $N = 93$. Post-hoc tests are presented for significant overall tests only. *p*-values were adjusted using the Benjamini-Hochberg correction and presented alongside the unadjusted *p*-values. $p < .05$ are set in bold. Analyses were conducted in multiply imputed data. Assessments took place before the intervention (T1) and subsequently at 4-week intervals (T2 – T4). ME = main effect; df = degrees of freedom.
