# Supplementary material for: Complementary Effect of an Educational Website for Children and Adolescents with Primary Headaches in Tertiary Care: A Randomized Controlled Trial
Source: Children (Basel). 2025 May 30;12(6):716. doi: 10.3390/children12060716 (PMC12190991; doi:10.3390/children12060716)
Supplement: Supplementary file 1 [file children-12-00716-s001.zip › S1 Used R Packages.pdf]

children

MDPI

Supplementary Materials S1. Used R Packages

- broom.mixed, version 0.2.9.4 [1]: Tidying outputs of multiply imputed data
- compareGroups, version 4.7.0 [2]: Group comparisons
- dplyr, version 1.1.1 [3]: Data wrangling
- easystats, version 0.7.3 [4]: Multilevel models and pooling of results
- EMAtools, version 0.1.4 [5]: Effect sizes of post-hoc tests
- ggmice, version 0.1.0 [6]: Plotting multiply imputed data
- haven, version 2.5.2 [7]: Importing and exporting files from SPSS
- janitor, version 2.2.0 [8]: Data cleaning and descriptive statistics
- lme4, version 1.1.33 [9]: Multilevel models
- lmerTest, version 3.1.3 [10]: Effect sizes
- mice, version 3.16.0 [11]: Multiple imputation using chained equations (mice)
- miceadds, version 3.17.44 [12]: Saving mice data
- mitools, version 2.4 [13]: Multiple imputation
- naniar, version 1.1.0 [14]: Missing data analysis
- nlme, version 3.1.157 [15]: Multilevel models
- patchwork, version 1.1.2 [16]: Graphs
- readxl, version 1.4.2 [17]: Data wrangling
- rstatix, version 0.7.2 [18]: Descriptive statistics
- scales, version 1.2.1 [19]: Graphs
- skimr, version 2.1.5 [20]: Data examination
- tidyverse, version 2.0.0 [21]: Data wrangling, analysis, and graphs
- writexl, version 1.4.2 [22]: Exporting results to Excel

References

1. Bolker B, Robinson D (2022) broom.mixed: Tidying Methods for Mixed Models. https://CRAN.R-project.org/package=broom.mixed
2. Subirana I, Sanz H, Vila J (2014) Building Bivariate Tables: The compareGroups Package for R. J Stat Soft 57:1–16
3. Wickham H, François R, Henry L et al. (2023) dplyr: A Grammar of Data Manipulation. https://CRAN.R-project.org/package=dplyr
4. Lüdecke D, Ben-Shachar MS, Patil I et al. (2022) easystats: Framework for Easy Statistical Modeling, Visualization, and Reporting. CRAN. https://doi.org/10.32614/CRAN.package.easystats
5. Kleiman E (2021) EMAtools: Data Management Tools for Real-Time Monitoring/Ecological Momentary Assessment Data. https://CRAN.R-project.org/package=EMAtools
6. Oberman H (2023) ggmice: Visualizations for 'mice' with 'ggplot2'. https://CRAN.R-project.org/package=ggmice
7. Wickham H, Miller E, Smith D (2023) haven: Import and Export 'SPSS', 'Stata' and 'SAS' Files. https://CRAN.R-project.org/package=haven
8. Firke S (2023) janitor: Simple Tools for Examining and Cleaning Dirty Data. https://CRAN.R-project.org/package=janitor
9. Bates D, Mächler M, Bolker B et al. (2015) Fitting Linear Mixed-Effects Models Using lme4. J Stat Soft 67:1–48. https://doi.org/10.18637/jss.v067.i01
10. Kuznetsova A, Brockhoff PB, Christensen RHB (2017) lmerTest Package: Tests in Linear Mixed Effects Models. J Stat Soft 82:1–26. https://doi.org/10.18637/jss.v082.i13
11. van Buuren S, Groothuis-Oudshoorn K (2011) mice: Multivariate Imputation by Chained Equations in R. J Stat Soft 45:1–67. https://doi.org/10.18637/jss.v045.i03
12. Robitzsch A, Grund S (2024) miceadds: Some Additional Multiple Imputation Functions, Especially for 'mice'. https://CRAN.R-project.org/package=miceadds
13. Lumley T (2019) mitools: Tools for Multiple Imputation of Missing Data. https://CRAN.R-project.org/package=mitools

14. Tierney N, Cook D (2023) Expanding Tidy Data Principles to Facilitate Missing Data Exploration, Visualization and Assessment of Imputations. J Stat Soft 105:1–31. https://doi.org/10.18637/jss.v105.i07
15. Pinheiro J, Bates D, R Core Team (2022) nlme: Linear and Nonlinear Mixed Effects Models. https://CRAN.R-project.org/package=nlme
16. Pedersen TL (2022) patchwork: The Composer of Plots. https://CRAN.R-project.org/package=patchwork
17. Wickham H, Bryan J (2023) readxl: Read Excel Files. https://CRAN.R-project.org/package=readxl
18. Kassambara A (2023) rstatix: Pipe-Friendly Framework for Basic Statistical Tests. https://CRAN.R-project.org/package=rstatix
19. Wickham H, Seidel DP (2022) scales: Scale Functions for Visualization. https://CRAN.R-project.org/package=scales
20. Waring E, Quinn M, McNamara A et al. (2022) skimr: Compact and Flexible Summaries of Data. https://CRAN.R-project.org/package=skimr
21. Wickham H, Averick M, Bryan J et al. (2019) Welcome to the tidyverse. Journal of Open Source Software 4:1686. https://doi.org/10.21105/joss.01686
22. Ooms J (2023) writexl: Export Data Frames to Excel 'xlsx' Format. https://CRAN.R-project.org/package=writexl

Disclaimer/Publisher's Note: The statements, opinions and data contained in all publications are solely those of the individual author(s) and contributor(s) and not of MDPI and/or the editor(s). MDPI and/or the editor(s) disclaim responsibility for any injury to people or property resulting from any ideas, methods, instructions or products referred to in the content.
